# Association between pupil dilation and implicit processing prior to object recognition via insight

**DOI:** 10.1038/s41598-018-25207-z

**Published:** 2018-05-02

**Authors:** Yuta Suzuki, Tetsuto Minami, Shigeki Nakauchi

**Affiliations:** 1 Department of Computer Science and Engineering, Toyohashi University of Technology, 1-1 Hibarigaoka Tempaku, Toyohashi, Aichi 441-8580 Japan; 2 Electronics-Inspired Interdisciplinary Research Institute, Toyohashi University of Technology, 1-1 Hibarigaoka Tempaku, Toyohashi, Aichi 441-8580 Japan

## Abstract

Insight refers to the sudden conscious shift in the perception of a situation following a period of unconscious processing. The present study aimed to investigate the implicit neural mechanisms underlying insight-based recognition, and to determine the association between these mechanisms and the extent of pupil dilation. Participants were presented with ambiguous, transforming images comprised of dots, following which they were asked to state whether they recognized the object and their level of confidence in this statement. Changes in pupil dilation were not only characterized by the recognition state into the ambiguous object but were also associated with prior awareness of object recognition, regardless of meta-cognitive confidence. Our findings indicate that pupil dilation may represent the level of implicit integration between memory and visual processing, despite the lack of object awareness, and that this association may involve noradrenergic activity within the locus coeruleus-noradrenergic (LC-NA) system.

## Introduction

When we recognize objects in daily life, our brains process the relevant visual information, regardless of whether we are conscious of this process. In particular, sudden insight—which refers to an instantaneous shift in comprehension during the perception of a stimulus, situation, event, or problem following a period of unconscious processing—allows us to obtain a new interpretation of our current situation, resulting in what is known as an “aha! moment” or the “eureka effect”. Beeman *et al*. observed that brain activity increased in the right anterior superior temporal gyrus when participants solved verbal problems using insight, relative to that observed when such problems were solved without insight^1,2^. Furthermore, several previous electroencephalography (EEG) studies have demonstrated that oscillatory activity within the alpha, beta, and gamma bands is significantly modulated by perceptual transitions during insight-based tasks^3–7^. Previous studies have examined the association between activity in specific brain regions and problem-solving at the precise moment of insight. However, although solutions may appear to present themselves instantaneously when derived via insight, several unconscious processing steps occur prior to recognition of the solution. Therefore, the goal of our study was to investigate whether processing prior to visual object recognition leads to subjective comprehension. Remarkably, Kounios *et al*. have indicated that the frequency of alpha-band activity in the right visual cortex increases 1.5 s prior to the recognition of solutions to verbal problems^8^. Additionally, Kietzmann *et al*. demonstrated that eye movement reflects distinct patterns of overt attention when participants are presented with an ambiguous object, suggesting that different eye movement patterns and neuronal mechanisms are at play prior to awareness of an ambiguous object^9^.

Over the past several decades, advancements in eye-tracking techniques have enabled researchers to efficiently and easily explore cognitive processes such as thought, memory, emotion, decision-making, and attention^10^. Although the pupil constricts under conditions of bright light and dilates under conditions of dim light^11^, several previous studies have indicated that cognitive factors such as cognitive effort, high working-memory load, and attentional state modulate the extent of pupil dilation, even at the preconscious level^12^.

Pupil dilation is controlled by the level of activation within the sympathetic nervous system, which is associated with noradrenergic activity in the locus coeruleus (LC). This sequence of phenomena is referred to as the locus coeruleus noradrenergic (LC-NA) system^13^. Additional studies have demonstrated that manipulation of memory processing using pharmacological agents leads to enhanced release of norepinephrine (NE)^14^. The pupillary response is also mediated by the activation of the superior colliculus (SC), the superficial layer of which receives input from the retina and mediates pupillary constriction via the Edinger–Westphal (EW) nucleus. The intermediate layer of the SC receives information from the regions of the frontal and parietal cortices that regulate attention shifting and motor input, and has been associated with the pupillary response via the EW nucleus^15^. We therefore hypothesized that pupil dilation is associated with subjective comprehension within regions involved in memory retrieval and attention shifting prior to the moment of insight, and that this association is modulated by activation of the sympathetic nervous system during insight-based problem solving.

As previously mentioned, the consolidation of information associated with an ambiguous object and memory processing may play an important role during object recognition. Smith *et al*. reported that eye movements are affected by the individual’s viewpoint of a familiar scene, and that awareness of the scene is determined by hippocampus-dependent memory^16^. Based on these findings, we hypothesized that the awareness characterized by memory-related processing of an ambiguous object is associated with prior, implicit object-recognition processing. We therefore employed an object-recognition task in which two durations of stimulus presentation were used to produce shifts in the state of object recognition among participants. Changes in pupil dilation were compared between recognition and non-recognition trials. We further investigated the association between pupil dilation and meta-cognitive processing by asking participants to rate their level of confidence in the recognition of each object. Pupillary responses were recorded during the presentation of stimuli, which consisted of ambiguous, transforming images composed of dots that had been set equal in luminance. In a follow-up experiment (Experiment 2), we investigated whether changes in pupil dilation were influenced by stimulus-specific features.

## Results

In order to monitor the pupillary response of present and subsequent comprehension when participants were given a problem-solving task involving movies containing ambiguous information, we conducted the experiment using two durations of stimulus presentation. Our findings indicated that pupillary dilation reflected not only an explicit change in the interpretation of the ambiguous movies, but also in the level of prior comprehension/predicted insight.

### Experiment 1

**Figure 1:**
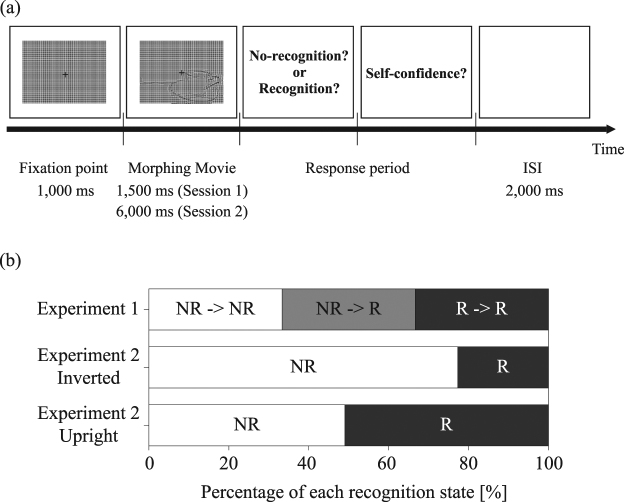
Experimental design and the percentage of each recognition state. (**a**) Protocol for Experiment 1. In each trial, a fixation point was presented for 1,000 ms prior to presentation of the stimulus. Each movie was presented for 1,500 ms in the first session and 6,000 ms in the second session. Each trial was separated by an inter-stimulus interval (ISI) of 2,000 ms. Participants reported whether they were able to recognize the contents of the movie following each trial using the keypad. (**b**) This percentage represents the averaged trial ratio of each recognition state among participants: P_NN_, P_NR_, and P_RR_ conditions. All trials consisted of 60 movies in Experiment 1 and 40 movies in Experiment 2. Therefore, 100% denotes the total number of trials for which responses were provided.

In Experiment 1, participants viewed identical moving images for 1,500 ms (Session 1) and 6,000 ms (Session 2). Participants were instructed to respond regarding whether they had recognized the contents of the movie, and to provide a rating of their confidence in their answer; the protocol is detailed in Fig. 1a. Prior to viewing the stimulus movies, participants were presented with a screen of homogeneous luminance, which was used to determine baseline pupillary responses. Participant responses at the end of each trial were categorized as follows: P_NN_: no-recognition (NR) to no-recognition (NR); P_NR_: no-recognition (NR) to recognition (R); and P_RR:_ recognition (R) to recognition (R). The average numbers of P_NN_, P_NR_, and P_RR_ trials were 21.9 ± 5.6, 22.4 ± 8.1, and 15.7 ± 6.0, respectively (mean ± SD). Figure 1b shows the percentage of each recognition state during Experiment 1. Object recognition was observed for 27% of stimuli in the first presentation session, while a lack of object recognition was observed for 37% of stimuli after the second, longer presentation session. In 36% of trials, object recognition was observed following the second session, but not after the first session. A one-way repeated-measures ANOVA revealed a significant main effect of the number of trials on object recognition (F(1,22) = 4.26, p = 0.0196). The number of recognition trials for the P_RR_ condition was significantly lower than that for the remaining two conditions. The average number of trials in each confidence condition (low, middle, high) for P_NR_ was 7.48 ± 3.76, 2.65 ± 2.48, and 10.26 ± 5.96, respectively (mean ± SD). The average number of trials in each confidence condition (low, middle, high) for P_NN_ were 5.22 ± 4.37, 1.96 ± 1.64, 11.87 ± 6.37, respectively (mean ± SD). The “middle” condition was excluded because the number of trials for this condition was significantly smaller than that for the other two conditions (p < 0.05). We therefore compared data of high and low confidence conditions to investigate whether confidence level was reflected in pupil diameter.

**Figure 2:**
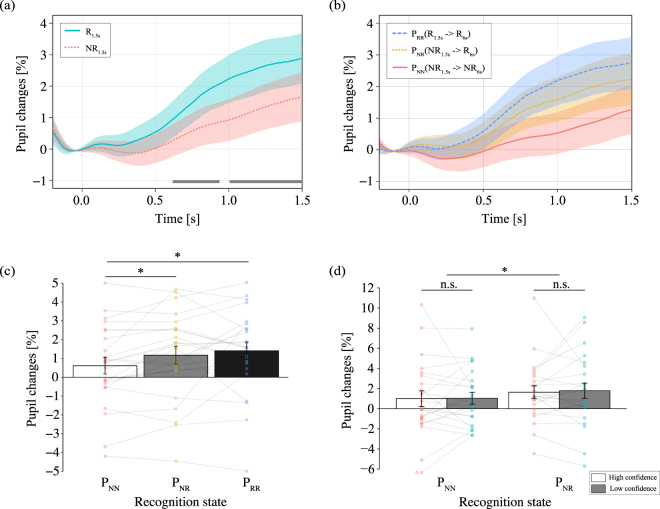
The time course of pupil responses based on recognition state. The horizontal axis indicates the time (in ms), while the vertical axis indicates the grand-averaged change in pupil dilation changes from baseline (from −200 ms to 0 ms). Shaded areas represent the standard error of the mean. The statistical significance of the comparisons is indicated by asterisks (*) for p < 0.05. (**a**) The blue line represents trials in which objects were recognized (R), while the red line represents trials in which objects were not recognized (NR). The gray bar represents the difference between R and NR trials; *P* values were corrected for multiple comparisons with an expected FDR of 0.05. (**b**) The grand-averaged time course of pupil changes during stimulus presentation for the first session (1,500 ms), classified based on subsequent insight during the second session (6,000 ms): no-recognition (NR) to no-recognition (NR), no-recognition (NR) to recognition (R), and recognition (R) to recognition (R). (**c**) The average pupil dilation from 0 ms to 1,500 ms for each recognition state. The colored circle indicates mean value for each participant data. (**d**) The average pupil dilation from 0 ms to 1,500 ms for each level of confidence according to subsequent changes in recognition state. The colored circle indicates mean value for each participant data.

Figure 2a shows the grand-averaged time course of changes in pupil dilation during stimulus presentation (1,500 ms) for the first session. T-tests revealed significant differences between conditions at each time course within the range of 658 ms to 918 ms and 1,031 ms to 1,500 ms after stimulus onset (p < 0.05; gray bar). *P* values were corrected for multiple comparisons with an expected false discovery rate (FDR) of 0.05. The average pupil dilation was also significantly greater for recognition trials than for non-recognition trials within the range of 0 ms to 1,500 ms (t(22) = 2.4395, p = 0.0228). This result suggests that even though the average luminance was strictly matched among the stimuli, pupil dilation was influenced by the recognition state. Figure 2b shows the grand-averaged time course of pupil changes during stimulus presentation (short: 1,500 ms) for the first session, classified based on subsequent insight during the second session (long: 6,000 ms) (e.g., P_NR_ indicates that participants recognized the object in the second session but not in the first session). Figure 2c shows the average pupil dilation from 0 ms to 1,500 ms for each cognitive condition indicated in Fig. 2b. A one-way repeated measures ANOVA was performed to compare pupil dilation among the three cognitive conditions (F(2,22) = 4.0203, p = 0.0246, ${{\rm{\eta }}}_{{\rm{p}}}^{2}$ = 0.0223). The results of this analysis revealed that pupil dilation was significantly greater in the P_RR_ and P_NR_ conditions than in the P_NN_ condition (t(22) = 2.8287, p = 0.0286 and t(22) = 2.4413, p = 0.0286, respectively). These findings indicated that pupil dilation reflects not only the subjective recognition state but also spontaneous neural activation in preparation for insight-based problem solving, in accordance with our hypothesis.

Previous studies have suggested that pupil dilation is associated with the level of confidence in object recognition^17,18^. Thus, we also analyzed our results based on level of confidence. Figure 2d shows the average pupil dilation from 0 ms to 1,500 ms for each level of reported confidence. A two-way repeated-measures ANOVA was conducted using pupil dilation in each cognitive condition and confidence level as factors. However, this analysis revealed a significant main effect between the P_RR_ and P_NR_ trials only (F(1,22) = 5.2730, p = 0.0316, ${{\rm{\eta }}}_{{\rm{p}}}^{2}$ = 0.0112). There were no main effects of confidence level or interaction effects (p = 0.8311). However, it is possible that the spatial distribution (e.g., low-frequency component) of each stimulus affects the pupillary response. We therefore conducted Experiment 2 to investigate external factors that contribute to pupil dilation.

### Experiment 2

**Figure 3:**
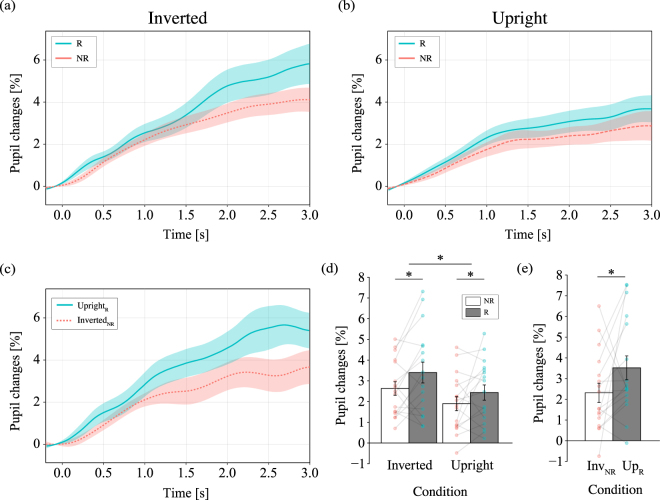
Pupil responses for inverted and upright versions of identical stimuli. The horizontal axis indicates the time (in ms), while the vertical axis indicates the grand-averaged change in pupil dilation changes from baseline (from −200 ms to 0 ms). Shaded areas represent the standard error of the mean. The statistical significance of the comparisons is indicated by asterisks (*) for p < 0.05. The grand-averaged time course of pupil changes during stimulus presentation for (**a**) the inverted session and (**b**) the upright session. The blue line represents trials in which objects were recognized (R), while the red line represents trials in which objects were not recognized (NR). (**c**) The grand-averaged pupil change classified based on response (recognition vs. non-recognition) to the identical stimulus movie in both the upright and inverted conditions. The pupil dilation was averaged from 0 ms to 3,000 ms during stimulus presentation. Analyses were performed for three recognition states: (**d**) R: recognition in both upright and inverted conditions, NR: non-recognition in both upright and inverted conditions, (**e**) Inv_NR_Up_R_: recognition in upright and non-recognition in inverted condition. The colored circle indicates mean value for each participant data.

In this experiment, we examined whether the spatial distribution of stimuli affects pupil dilation in order to eliminate the possibility of its potential involvement in pupillary response in Experiment 1. Participants were presented with inverted (Session 1) and upright moving images (Session 2) for 3,000 ms. Participants were instructed to respond regarding whether they had recognized the contents of the movie. Figure 3a,b show the grand-averaged time course of changes in pupil dilation classified based on response (recognition vs. non-recognition) from 0 ms to 3,000 ms during stimulus presentation in both the inverted and upright conditions. The greater extent of pupil dilation observed in the inverted condition may have been associated with the increased cognitive effort required for mental rotation of the stimulus movie. Therefore, we performed bias-correction for the pupillary response in the upright condition (details of the analysis are stated in the Methods section). Figure 3c shows the grand-averaged time course of changes in the bias-corrected pupil dilation. This may reveal the effects of spatial distribution of the stimuli on pupil dilation for the same stimulus movies but in different recognition states were compared. These results are summarized in Fig. 3d,e.

Figure 3d shows the average changes in pupil dilation from 0 ms to 3,000 ms for each cognitive condition depicted in Fig. 3a,b and e shows the average changes in the bias-corrected pupil dilation depicted in Figure 3c. A two-way repeated measures ANOVA was performed to evaluate pupil dilation according to each cognitive condition and orientation condition shown in Fig. 3d. We identified significant effects of recognition state (F(1,16) = 6.2149, p = 0.0240, ${{\rm{\eta }}}_{{\rm{p}}}^{2}$ = 0.0391) and orientation (F(1, 16) = 11.0267, p = 0.0043, ${{\rm{\eta }}}_{{\rm{p}}}^{2}$ = 0.0660) on pupil dilation. These results showed that a lager pupil dilation was observed for the recognition condition than for non-recognition trials within the range of 0 ms to 3,000 ms, in accordance with the findings of Experiment 1. T-tests revealed that the bias-corrected pupillary response in Fig. 3e was significantly greater for recognized objects than for non-recognized objects (t(16) = 4.8928, p = 0.0419, ${{\rm{\eta }}}_{{\rm{p}}}^{2}$ = 0.1129). The results indicate that increased pupillary response to identical stimulus movies reflects the cognitive state. Thus, stimulus properties such as spatial distribution are unlikely associated with pupil dilation in the current study.

**Figure 4:**
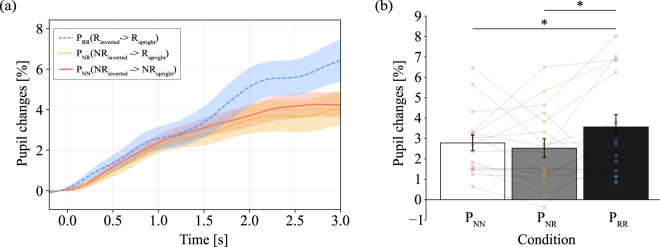
Pupil responses based on recognition state for inverted and upright. The horizontal axis indicates the time (in ms), while the vertical axis indicates the grand-averaged change in pupil dilation changes from baseline (from −200 ms to 0 ms). Shaded areas represent the standard error of the mean. The statistical significance of the comparisons is indicated by asterisks (*) for p < 0.05. (**a**) The grand-averaged time course of pupil changes during stimulus presentation for the inverted condition, classified based on subsequent insight during the upright condition: no-recognition (NR) to no-recognition (NR), no-recognition (NR) to recognition (R), and recognition (R) to recognition (R). (**b**) The average pupil dilation from 0 ms to 3,000 ms for each recognition state. The colored circle indicates mean value for each participant data.

Figure 4a shows the grand-averaged time course of pupil changes during stimulus presentation for the inverted condition, classified based on subsequent cognition during the upright condition (e.g., P_NR_ indicates that participants recognized the object in the upright condition but not in the inverted condition). Figure 4b shows the average pupil dilation from 0 ms to 3,000 ms for each cognitive condition indicated in Fig. 4a. A one-way repeated measures ANOVA was performed to compare pupil dilation among the three cognitive conditions (F(2,16) = 6.4464, p = 0.0042, ${{\rm{\eta }}}_{{\rm{p}}}^{2}$ = 0.1063). The results of this analysis revealed that pupil dilation was significantly greater in the P_RR_ condition than in the P_NR_ and P_NN_ conditions (t(16) = 3.0874, p = 0.0067 and t(16) = 2.7119, p = 0.0148, respectively). These results showed that the pupillary response in the inverted condition did not predict the recognition state in the upright condition, which was contrary the results of Experiment 1. This incongruence of P_NR_ indicated that when the participants attempted to interpret an object in the inverted and upright conditions, a memory processing mechanism distinct from that operating during Experiment 1 is associated with the insight-based recognition.

## Discussion

In the present study, we investigated the temporal dynamics of pupil dilation in order to elucidate the potential mechanisms underlying insight-based recognition of ambiguous objects. We hypothesized that pupil dilation reflects not only the subjective recognition state but also spontaneous neural activation in preparation for insight-based problem solving. Our findings indicated that, even when participants were unable to recognize the object in the movie, pupil dilation was associated with subsequent comprehension. Moreover, such responses were not associated with motor preparation to respond affirmatively^19,20^, as some participants reported that they had not recognized the objects in either the P_NR_ or P_NN_ conditions. In Experiment 2, we verified that the spatial distribution of each stimulus (e.g., low-frequency components) did not produce effects on pupil dilation in each cognitive condition based on responses to identical stimuli in upright and inverted conditions. The results of this experiment suggest that pupil dilation reflects pre-conscious processing and implicit memory retrieval prior to the recognition of ambiguous objects.

Several previous cognitive studies have demonstrated that corresponding pupillary responses follow both instantaneous and sustained alterations in LC activity^13,21–24^. In humans, pupil dilation is associated with the exploitation response via phasic mode activity following instantaneous LC activation during task-evoked processing. Our finding that pupil dilation was greater for recognition trials than non-recognition trials may reflect increases in LC activity due to task engagement, memory recall, and problem-solving processes. These results suggest that pupil dilation can be used as an index of transition between cognitive states when insight regarding previously ambiguous objects has been attained.

Several previous studies have investigated this phenomenon and its underlying neural mechanisms using the Remote Associates Test, which requires participants to provide verbal solutions to word-association problems^2,25–27^. Additional research has suggested that specific alterations in alpha-band activity (8-12 Hz) in the medial frontal and temporal regions—which are associated with cognitive control and semantic processing, respectively—facilitate problem solving via insight prior to the presentation of verbal problems^8,28^. In this previous fMRI study, activation of the anterior cingulate cortex (ACC) was observed prior to problem-solving. Activation of the ACC is characterized by the suppression of extraneous thoughts, tactic searching, and shifts in attention, while increased alpha-band activity has been associated with processing in the occipital, superior temporal, inferior frontal, and cingulate cortices^29^. Notably, the cingulate cortex exhibits projections to the LC^22^. Several studies have indicated that pupil dilation varies depending on the level of activation within the LC-NA system and reflects numerous cognitive processes such as memory retrieval, subjective perception, and decision-making^30–33^. Therefore, the significant increase in pupil dilation observed during recognition trials may reflect visual memory processes associated with the processing of ambiguous visual stimuli via the LC-NA system.

If pupil dilation indeed reflects spontaneous visual memory processes, as our findings suggest, the level of dilation can then be used to predict the likelihood of successful retrieval, as previous studies have suggested^34,35^. In accordance with this hypothesis, several studies have demonstrated that novel scenes elicit greater pupil constriction than familiar scenes^36^, and that familiarity with visual stimuli is significantly associated with the level of pupil dilation^37,38^. Furthermore, memory retrieval following the presentation of contextual cues is processed in the prefrontal cortex and hippocampus^39^, a process mediated by LC activity^40^. Thus, these findings imply that the interaction between the hippocampus and prefrontal cortex activates the sympathetic nervous system via the LC-NA system, reflecting the integration of memory and pre-conscious visual processing.

In the present study, we further investigated the association between pupil dilation and meta-cognitive factors of cognitive confidence by asking participants to rate their level of confidence in each response. Despite the findings of previous studies regarding meta-cognitive confidence during decision-making or memory strength^17,41^, we did not observe a meta-cognitive effect of confidence level on object recognition. In contrast, Lempert *et al*. reported a negative association between confidence in one’s responses and pupil dilation in the post-decision period^18^. This discrepancy may be explained by the finding that high confidence in object recognition promotes pupillary response, while low confidence related to the uncertainty is also associated with pupil dilation. Consequently, although meta-cognition during the decision-making period may influence the pupillary response, differences in pupillary dilation may occur along with subsequent changes in the recognition state, even among participants with high or low confidence in their responses.

We also examined other factors known to influence pupil dilation using inverted and upright versions of identical stimuli (Experiment 2). We observed an effect of mental rotation on pupillary dilation as well as cognitive state. As observed for short-term memory tasks^30^, mental rotation causes linear increases in reaction times during object match/mismatch tasks^42^. However, although pupil dilation was associated with mental rotation in the inverted condition, this effect was canceled by comparing non-recognition trials between the two orientation conditions. As significant differences in pupil dilation to the identical stimulus movie in Experiment 2 were observed between the recognition and non-recognition trials, our findings suggest that pupil responses were not affected by stimulus-specific features. Our additional analysis of the pupillary response classified by P_RR_, P_NR_, or P_NN_ during the inverted condition did not predict the later behavioral response. This implies that the consecutive object movie in Experiment 1 is important to predict the later behavior in relation to the memory processing. When we recognize an object, the recognition is affected by geometric construction such as shape, orientation, and spatial distribution of the object^43^. In Experiment 2, the inverted movie may be interpreted as a pattern of an object distinct from that in the upright condition, whereas the pupillary prediction effect of P_NR_ accompanied by the implicit memory processing in Experiment 1 may be involved with the expectation as an object constructed by moving dots.

The present study possesses some limitations of note. We asked the participants whether they had recognized the contents of the movie, although we did not ask them to provide the answer with which they associated the movie. Thus, we were unable to determine whether participants correctly recognized the movie contents. However, our results cannot be explained by a yes/no response^33^ because the pupil was dilated in the P_NR_ condition, even when participants responded “no” in the P_NN_ condition. Moreover, we analyzed the data based on the average values in each recognition condition because we were unable to determine the precise moment at which insight occurred. In future studies, a model of the pupillary response during a single trial should be estimated based on the time at which insight occurred.

In conclusion, our findings demonstrate that pupil dilation reflects both explicit recognition of ambiguous objects and implicit processing prior to insight. The present study is the first to report the association between increases in pupillary dilation and an instantaneous shift in object recognition. However, changes in pupil dilation reflected object recognition, even in participants who provided low confidence ratings regarding high confidence and uncertainty. Our findings suggest that pupil dilation is influenced by the transitions from exploration- to exploitation-related, task-engagement phasic activity within the LC-NA system. Moreover, such prior pupil dilation may represent the integration of memory and the pre-conscious processing of ambiguous visual information. Thus, the present study is the first to characterize pupil dilation as an index of implicit visual processing/object recognition prior to awareness.

## Methods

### Participants

All experimental procedures were in accordance with the ethical principles outlined in the Declaration of Helsinki and approved by the Committee for Human Research at the Toyohashi University of Technology, and the experiment was strictly conducted in accordance with the approved guidelines of the committee. All participants provided written informed consent. Twenty-six volunteers (17 men, 9 women; age range: 20–25 years) took part in Experiment 1. Twenty volunteers (10 men, 10 women; age range: 20–25 years) took part in Experiment 2. Three participants in each experiment were excluded from pupil analyses due to eye blink on more than 30% of trials. All participants were undergraduate and graduate students with normal or corrected-to-normal vision.

### Stimuli and experimental procedure

#### Experiment 1

Figure 1a shows the protocol for Experiment 1. The experiment consisted of 60 movies and was conducted over two sessions. In these two sessions, the 60 stimuli were identical, and each stimulus was presented only one time within each session in random order. In each trial, a fixation point was presented for 1,000 ms prior to presentation of the stimulus. Each movie was presented for 1,500 ms in the first session and 6,000 ms in the second session. Each trial was separated by an inter-stimulus interval (ISI) of 2,000 ms. Participants reported whether they were able to recognize the contents of the movie following each trial using the keypad.

**Figure 5:**
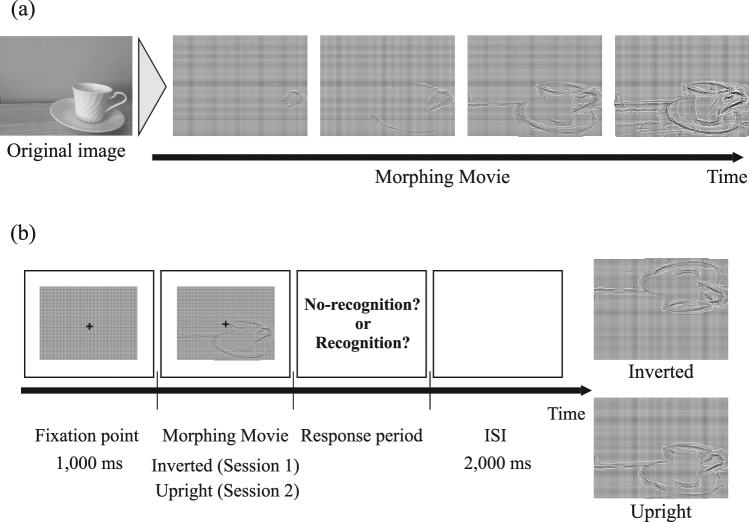
Experimental paradigm and stimulus. (**a**) Overview of the stimulus representation. Ambiguous movies were generated by the deformation of dots calculated from the original image. Stimuli consisted of 60 movies of non-living objects (e.g., cup). (**b**) Protocol for Experiment 2. In each trial, the fixation point was presented for 1,000 ms, following which the stimulus was presented for 3,000 ms. Each trial was separated by an inter-stimulus interval (ISI) of 2,000 ms. Participants reported whether they were able to recognize the contents of the movie following each trial using the keypad.

Stimuli consisted of 60 movies of non-living objects (e.g., cup as shown in Fig. 5a) derived using the “Dots method”^44^. The spatial complexity and difficulty of the task were controlled by manipulating three object-related parameters, as follows. The elastic constant and distance were set to *K* = 10 and *D* = 5, respectively, in accordance with methods described by Moca *et al*.^44^. The gravitational constant was manipulated linearly from 0.06 to 0.186 in 180 steps. A logistic function was used to manipulate the speed of each movie from 1 to 720 frames. The speed of each movie was identical and did not depend on the duration of presentation. Thus, in the first session, the movie ended prior to completion of 180 steps.

The background luminance was set to 60.07 cd/m^2^ for all movies to avoid the effects of differences in object luminance on pupil dilation. The outline of each image was transformed into a series of dots (0.16 cd/m^2^). Luminance of the stimuli was calibrated using a chromameter (ColorCAL II, Cambridge Research System, Kent, UK). Participants rested for at least 5 min between each session. A nine-point calibration was performed prior to each session, following which each session was conducted for approximately 10 min.

All stimuli were presented at a visual angle of 16.40 degrees × 12.45 degrees at the center of a liquid-crystal display (LCD) monitor (Viewpixx3D, VPixx Technologies, Saint-Bruno-de-Montarville, QC, Canada) with a resolution of 1920 × 1080 and refresh rate of 120 Hz. The dot size was set to 0.07 degrees, and the fixation point was located 0.3 degrees from the center. Each participant’s chin was fixed at a viewing distance of 600 mm. The task was conducted in a shielded darkroom and executed in MATLAB2014b (The MathWorks, Natick, MA, USA) using Psychtoolbox 3^45^.

Participants were instructed to respond regarding whether they had recognized the contents of the movie using the keypad, and to provide a rating of their confidence in their answer. When participants responded that they had recognized the object (“yes”), confidence was rated along a three-point scale (high, middle, and low confidence) ranging from high confidence of “very sure” to low confidence of “not sure”. When participants responded that they had not recognized the object (“no”), confidence was rated along a three-point scale (high, middle, and low confidence) ranging from high confidence of “no comprehension” to low confidence of “likely to comprehend”. In this case, high confidence indicated that they could not recognize the object, and low confidence indicated that they would be likely to find something familiar in the ambiguous movie but could not recognize the object.

### Experiment 2

The protocol for Experiment 2 is presented in Fig. 5b. The experiment consisted of 60 movies and was conducted over two sessions. Participants rested for more than 5 min between each session. In these two sessions, the 60 stimuli were identical but differed from those of Experiment 1, and each stimulus was presented only one time within each session in random order. However, inverted images were presented in the first session, while upright images were presented in the second session. In each trial, the fixation point was presented for 1,000 ms, following which the stimulus was presented for 3,000 ms. Each trial was separated by an ISI of 2,000 ms. Participants reported whether they were able to recognize the contents of the movie following each trial using the keypad. A nine-point calibration was performed prior to each session, following which each session was conducted for approximately 8 min.

### Recording and analysis of pupil size

Pupil size and eye movement during the task were measured using an eye-tracking system (EyeLink 1000, SR Research, Oakland, Canada) at a sampling rate of 500 Hz. Movement of the participant’s left eye was recorded using an infrared light video camera at a resolution of no more than 0.1°. The eye blinks were interpolated using cubic-spline interpolation^46^. Trials with additional artifacts, found using peak change on the velocity of the pupil response, were excluded from the analysis (average rejected trials were 3.7 ± 2.9 trials per participant). Pupil size was generated by the device in arbitrary units. In the time course analysis, the pupil size at stimulus onset in each trial was normalized relative to the baseline pupil size, following which smoothing of each data point with ±5 sampling points and a band-stop filter (cut-off: 6–10 Hz) were performed. Baseline pupil size was computed as an average of data collected prior to stimulus onset (movie presentation), from −200 ms to 0 ms (presentation onset). The baseline corrected averaged pupil size from the presentation period of 0 ms to 1,500 ms in Experiment 1 and 3,000 ms in Experiment 2 were compared for each condition. In Experiment 2, we observed a significant difference in pupil dilation between the inverted and upright condition, which may have been associated with the level of cognitive effort required for mental rotation. Therefore, we subtracted the pupillary response for the same stimulus movies in the non-recognition trials in the inverted condition from that in the upright condition in order to align the data with the baseline pupillary response. After this manipulation, the biased pupillary response was added to that of the upright condition in order to evaluate changes in pupil dilation due to the cognitive state.

### Statistical analysis

In Experiment 1, participant responses at the end of each trial were categorized into three cognitive conditions as follows: P_NN_: no-recognition (NR) to no-recognition (NR); P_NR_: no-recognition (NR) to recognition (R); and P_RR:_ recognition (R) to recognition (R). In the time course of pupil diameter changes, the significant differences were corrected with FDR for multiple comparisons using the Benjamini and Hochberg method^47^. We also calculated the grand-averaged change in pupil dilation from baseline (from −200 ms to 0 ms). A one-way repeated-measures analysis of variance (ANOVA) was conducted using the cognitive conditions as factors. Two-way ANOVAs were performed using pupil dilation in each recognition state and confidence level as factors. T-tests were used to compare the mean pupil dilation between recognition and non-recognition states.

In Experiment 2, two-way ANOVAs were performed using pupil dilation in each recognition state and orientation (i.e., inverted or upright) as factors. The level of statistical significance was set to p < 0.05 for all analyses. Pairwise comparisons for main effects were corrected for multiple comparisons using the Bonferroni method. Effect sizes (partial η^2^; ${{\rm{\eta }}}_{{\rm{p}}}^{2}$) were determined for the ANOVA. Greenhouse-Geisser corrections were performed when the results of Mauchly’s sphericity test were significant.

### Data availability

Participant data sets and experimental scripts for analysis are available from github (https://github.com/suzuki970/Experimental_data/tree/master/P02).
